# Supplementary material for: Severe postpartum haemorrhage at a large referral hospital in Uganda: A prospective observational pilot study
Source: PLoS One. 2025 Sep 3;20(9):e0331512. doi: 10.1371/journal.pone.0331512 (PMC12407487; doi:10.1371/journal.pone.0331512)
Supplement: S1 Table — (DOCX) [file pone.0331512.s001.docx]

|  | In-house (N=13)  n (%) | Referral (N=47)  n (%) | Overall (N=60)  n (%) |
| --- | --- | --- | --- |
| Systolic blood pressure < 80mm Hg and pulse ≥ 120 bpm | 3 (23.1) | 25 (53.2) | 28 (46.7) |
| Systolic blood pressure < 80mm Hg | 8 (61.5) | 32 (68.1) | 40 (66.7) |
| Shock (≥ 1 of the above fulfilled) | 9 (69.2) | 39 (83.0) | 48 (80.0) |
| Administration of vasoactive drugs | 3 (23.1) | 13 (27.7) | 16 (26.7%) |
| Cardiovascular dysfunction (shock and/or vasoactive drugs) | 10 (76.9) | 42 (89.4) | 52 (86.7) |
