# Supplementary material for: Severe postpartum haemorrhage at a large referral hospital in Uganda: A prospective observational pilot study
Source: PLoS One. 2025 Sep 3;20(9):e0331512. doi: 10.1371/journal.pone.0331512 (PMC12407487; doi:10.1371/journal.pone.0331512)
Supplement: S2 Table — (DOCX) [file pone.0331512.s002.docx]

|  | In-house (N=13)  n (%) | Referral (N=47)  n (%) | Overall (N=60)  n (%) |
| --- | --- | --- | --- |
| ICU admission | 5 (38.5) | 9 (19.1) | 14 (23.3) |
| Laparotomy (hysterectomy and/or B-lynch) | 1 (7.7) | 13 (27.7) | 14 (23.3) |
| Blood transfusion | 10 (76.9) | 37 (78.7) | 47 (78.3) |
| Critical intervention (≥ 1 of the above fulfilled) | 10 (76.9) | 37 (78.7) | 47 (78.3) |
