# Supplementary material for: Severe postpartum haemorrhage at a large referral hospital in Uganda: A prospective observational pilot study
Source: PLoS One. 2025 Sep 3;20(9):e0331512. doi: 10.1371/journal.pone.0331512 (PMC12407487; doi:10.1371/journal.pone.0331512)
Supplement: S3 Table — (DOCX) [file pone.0331512.s003.docx]

|  | In-house (N=13)  n (%) | Referral (N=47)  n (%) | Overall (N=60)  n (%) |
| --- | --- | --- | --- |
| **Additional oxytocin 20 IU given** |  |  |  |
| No | 6 (46.2) | 16 (34.0) | 22 (36.7) |
| Yes | 2 (15.4) | 17 (36.2) | 19 (31.7) |
| Atony or retained products not the cause | 5 (38.5) | 14 (29.8) | 19 (31.7) |
| **Additional carboprost given** |  |  |  |
| No | 6 (46.2) | 25 (53.2) | 31 (51.7) |
| Yes | 2 (15.4) | 8 (17.0) | 10 (16.7) |
| Atony or retained products not the cause | 5 (38.5) | 14 (29.8) | 19 (31.7) |
