# Supplementary material for: Severe postpartum haemorrhage at a large referral hospital in Uganda: A prospective observational pilot study
Source: PLoS One. 2025 Sep 3;20(9):e0331512. doi: 10.1371/journal.pone.0331512 (PMC12407487; doi:10.1371/journal.pone.0331512)
Supplement: S4 Table — (DOCX) [file pone.0331512.s004.docx]

|  | In-house (N=13)  n (%) | Referrals (N=47)  n (%) | Overall (N=60)  n (%) |
| --- | --- | --- | --- |
| **Previous CS** |  |  |  |
| No previous CS | 10 (76.9) | 32 (68.1) | 42 (70.0) |
| At least 1 previous CS | 3 (23.1) | 15 (31.9) | 18 (30.0) |
| **Any pre-existing conditions** |  |  |  |
| No | 10 (76.9) | 42 (89.4) | 52 (86.7) |
| Yes | 3 (23.1) | 5 (10.6) | 8 (13.3) |
| **Complications in previous pregnancies** |  |  |  |
| No previous complications | 8 (61.5) | 34 (72.3) | 42 (70.0) |
| Complications | 5 (38.5) | 13 (27.7) | 18 (30.0) |
| **ANC attendance** |  |  |  |
| Not attended | 0 (0) | 1 (2.1) | 1 (1.7) |
| Attended | 13 (100) | 46 (97.9) | 59 (98.3) |
| **Antenatal problems in current pregnancy** |  |  |  |
| No | 8 (61.5) | 32 (68.1) | 40 (66.7) |
| Yes | 5 (38.5) | 15 (31.9) | 20 (33.3) |
