# Supplementary material for: Severe postpartum haemorrhage at a large referral hospital in Uganda: A prospective observational pilot study
Source: PLoS One. 2025 Sep 3;20(9):e0331512. doi: 10.1371/journal.pone.0331512 (PMC12407487; doi:10.1371/journal.pone.0331512)
Supplement: S5 Table — (DOCX) [file pone.0331512.s005.docx]

|  | In-house (N=13)  n (%) | Referrals (N=47)  n (%) | Overall (N=60)  n (%) |
| --- | --- | --- | --- |
| Preeclampsia | 1 (7.7) | 1 (2.1) | 2 (3.3) |
| Eclampsia | 0 (0) | 2 (4.3) | 2 (3.3) |
| Fever during labour | 0 (0) | 1 (2.1) | 1 (1.7) |
| Foetal distress | 0 (0) | 4 (8.5) | 4 (6.7) |
| Uterine rupture | 0 (0) | 9 (19.1) | 9 (15.0) |
| Cord prolapse | 0 (0) | 0 (0) | 0 (0) |
| Polyhydramnionous | 0 (0) | 0 (0) | 0 (0) |
| Bleeding/placenta praevia | 4 (30.8) | 18 (38.3) | 22 (36.7) |
| Shoulder dystocia | 0 (0) | 0 (0) | 0 (0) |
|  |  |  |  |
