# Supplementary figures and images for: Severe postpartum haemorrhage at a large referral hospital in Uganda: A prospective observational pilot study
Source: PLoS One. 2025 Sep 3;20(9):e0331512. doi: 10.1371/journal.pone.0331512 (PMC12407487; doi:10.1371/journal.pone.0331512)

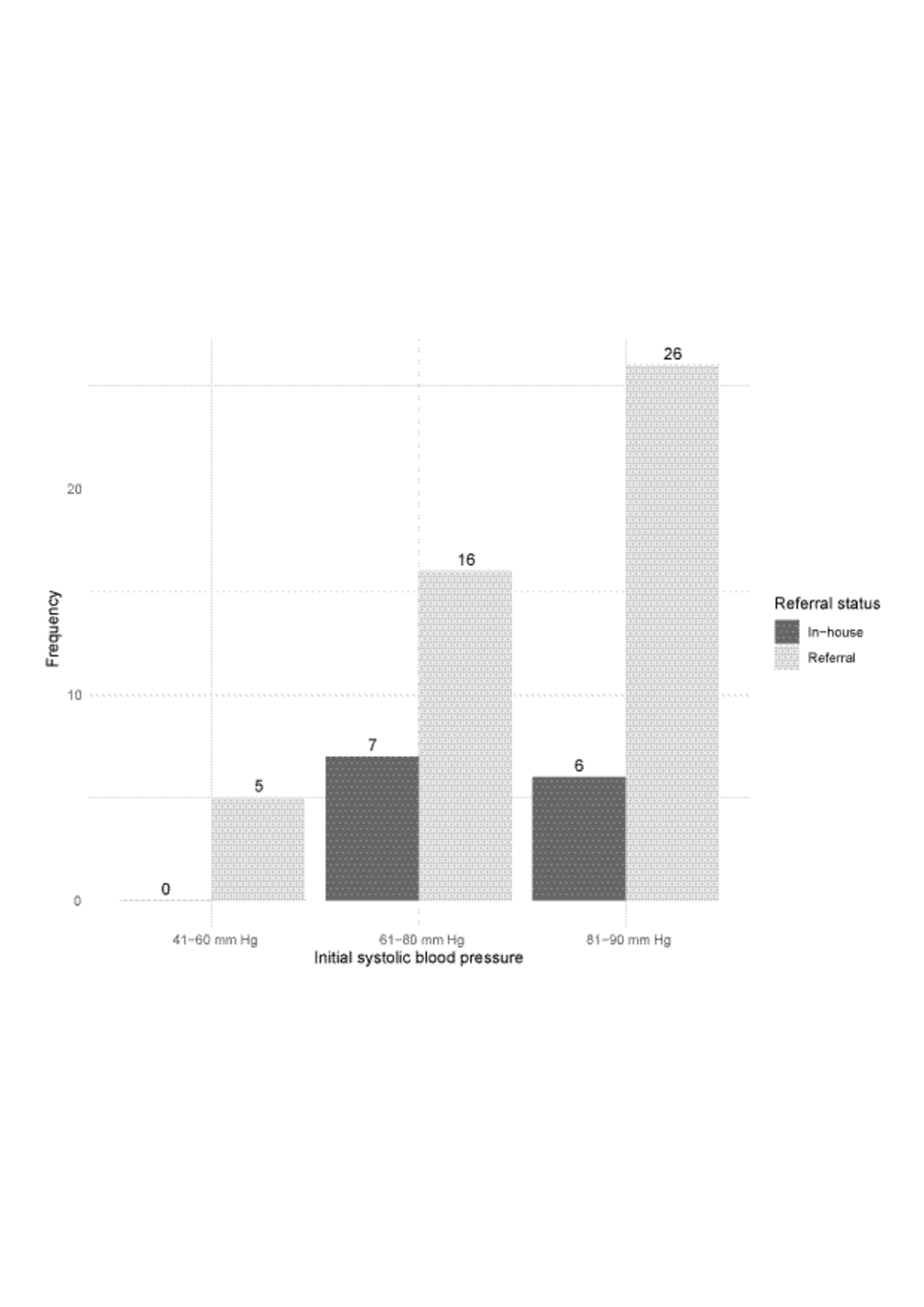

Supplement: S1 Fig — (TIF) [file pone.0331512.s006.tif]

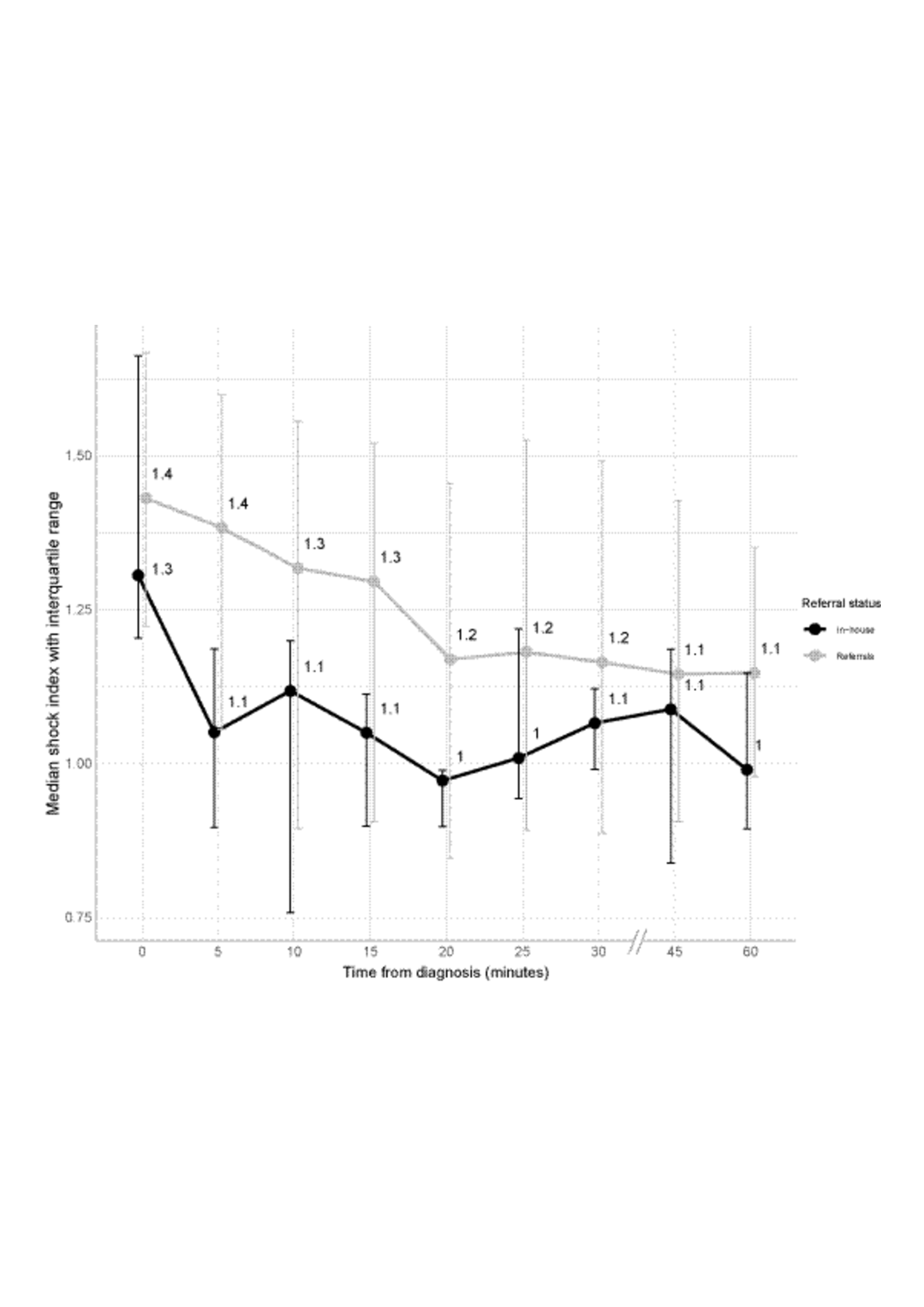

Supplement: S2 Fig — (TIF) [file pone.0331512.s007.tif]

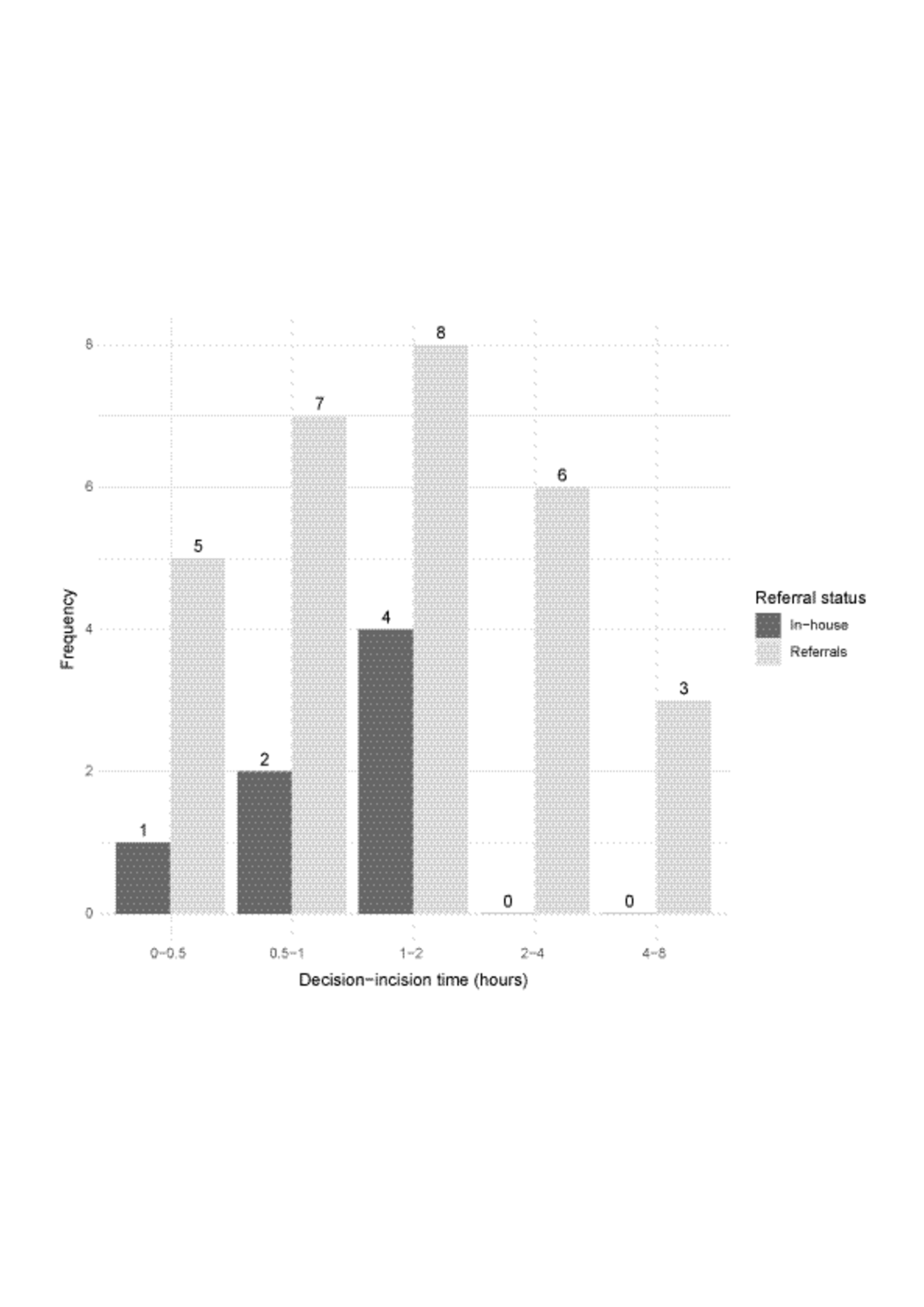

Supplement: S3 Fig — (TIF) [file pone.0331512.s008.tif]

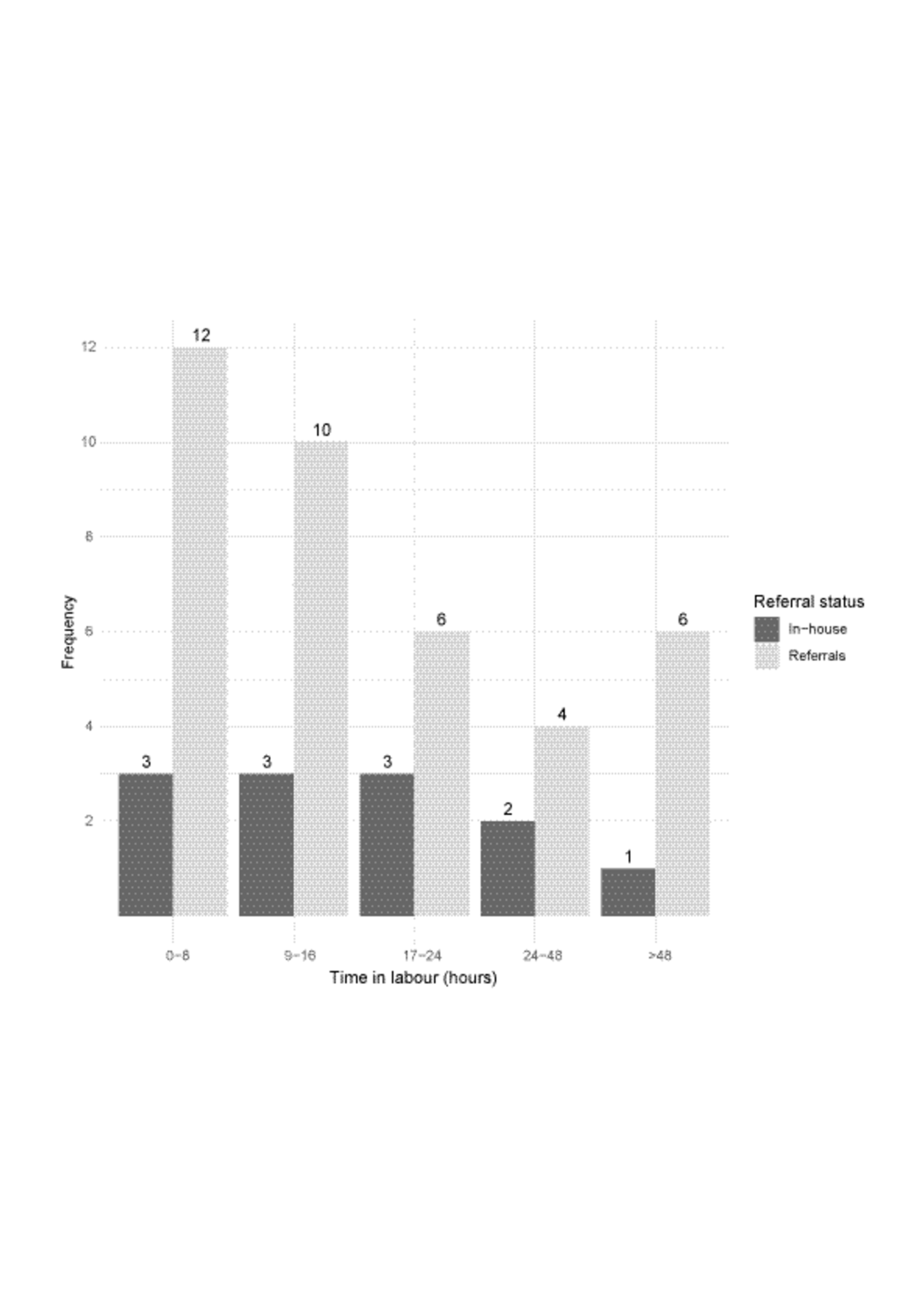

Supplement: S4 Fig — (TIF) [file pone.0331512.s009.tif]

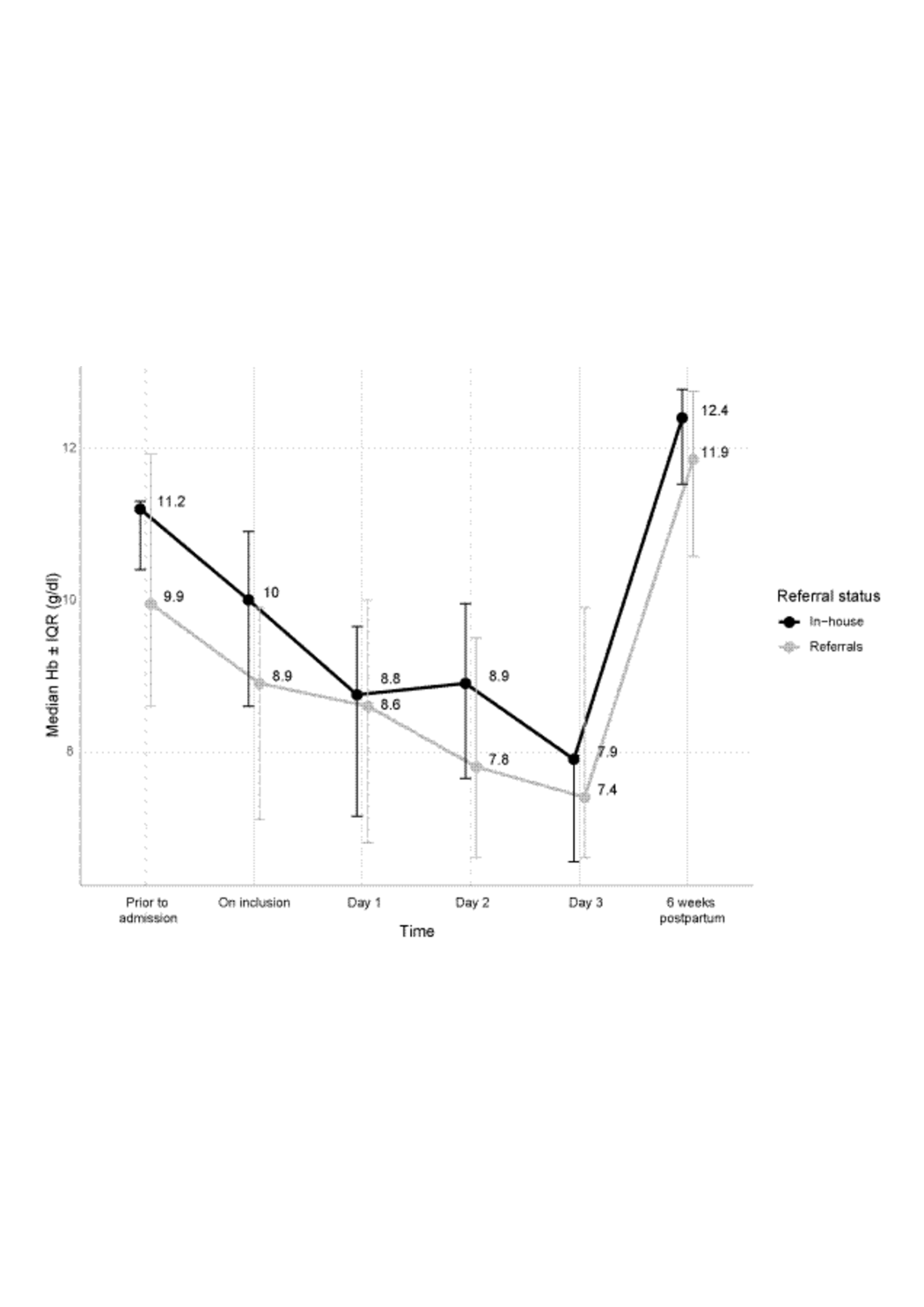

Supplement: S5 Fig — (TIF) [file pone.0331512.s010.tif]

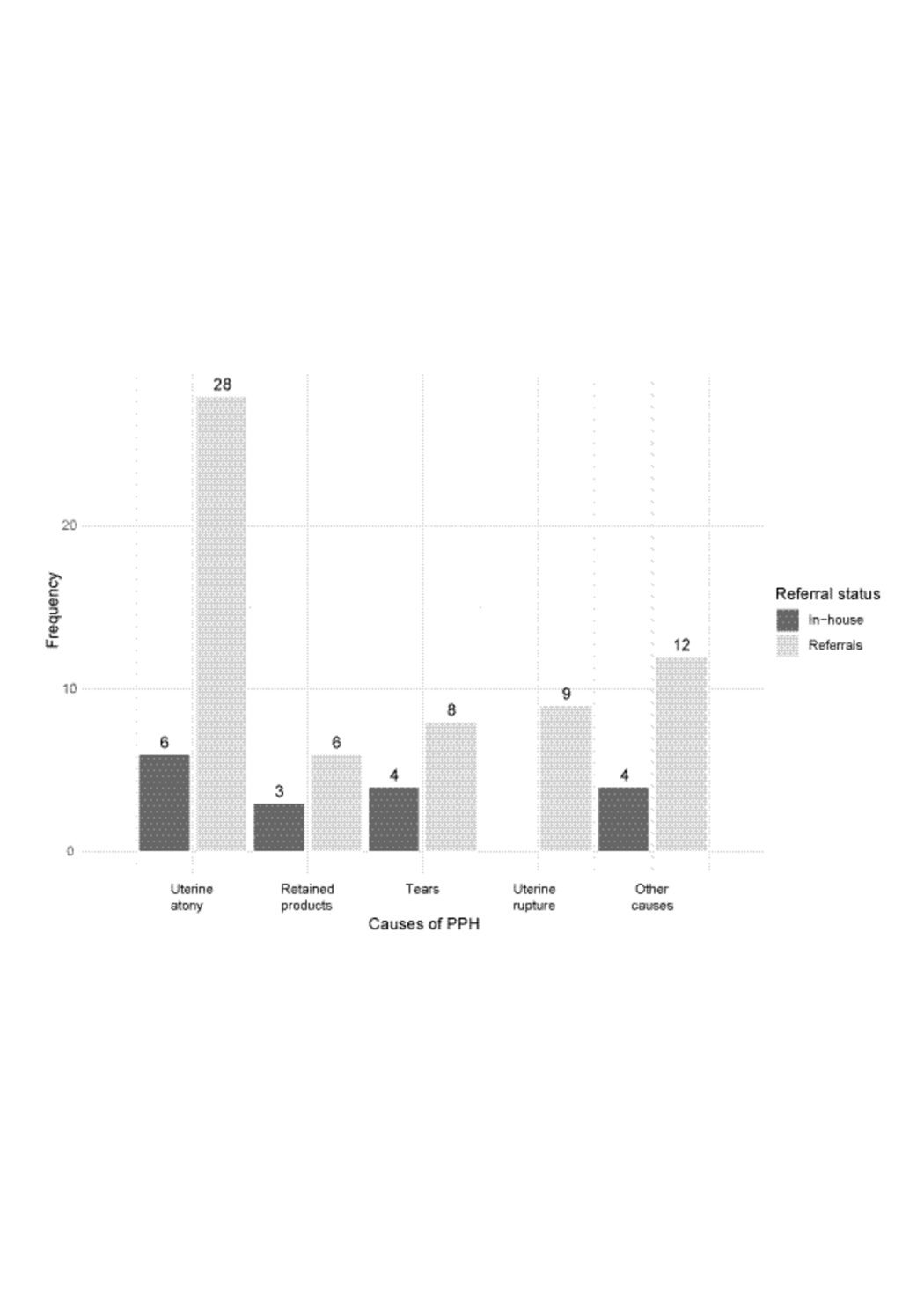

Supplement: S6 Fig — Examples of other causes include placental abruption, placenta accreta spectrum, uterine tears and coagulopathies. (TIF) [file pone.0331512.s011.tif]
