# Supplementary material for: Severe postpartum haemorrhage at a large referral hospital in Uganda: A prospective observational pilot study
Source: PLoS One. 2025 Sep 3;20(9):e0331512. doi: 10.1371/journal.pone.0331512 (PMC12407487; doi:10.1371/journal.pone.0331512)
Supplement: Attachment — Submitted filename: recenzja PLOS.docx [file pone.0331512.s013.docx]

Review of the Manuscript on Postpartum Hemorrhage Management at Kawempe National Referral Hospital

This manuscript addresses a critical public health issue — postpartum hemorrhage (PPH), which remains the leading cause of maternal mortality globally, particularly in low-income countries. The authors effectively highlight the significance of PPH as a major contributor to maternal deaths and severe maternal morbidity, particularly in Uganda, where PPH is responsible for approximately 35% of maternal deaths. The focus on Kawempe National Referral Hospital (KNRH) — Uganda’s largest obstetric referral hospital — is highly relevant, as this facility receives numerous emergency cases, including severe PPH. By investigating adherence to national guidelines, the study provides valuable insights into the quality of care delivered in this high-risk setting.

The study is well-prepared and presents significant findings that have the potential to inform both clinical practice and policy improvements. However, I suggest minor revisions to enhance clarity and ensure the manuscript’s completeness.

Suggested Revisions and Clarifications

Clarification of Acronym (DHS)

In line 6, the abbreviation DHS is mentioned for the first time without explanation. To ensure the readership understands its meaning, I recommend defining this acronym in its first use (e.g., Demographic and Health Survey).

Route of Misoprostol Administration

The manuscript references the use of misoprostol, but it is unclear which route of administration was employed — buccal, vaginal, or rectal. Given that the route of administration may significantly influence the drug’s efficacy and absorption rate, I suggest providing this detail.

Overall Assessment

This is a well-prepared manuscript that addresses an important and timely topic. The authors provide valuable insights into the challenges associated with managing severe PPH at KNRH, with a particular focus on adherence to national guidelines. The findings have important implications for improving the quality of obstetric care and reducing maternal morbidity and mortality in Uganda and other low-resource settings.

With the above minor revisions and clarifications, this manuscript will strongly contribute to the literature on maternal health. I commend the authors for their thorough work and thoughtful analysis.

Recommendation: Minor revisions are required.

Sincerely,
