# Supplementary material for: Severe postpartum haemorrhage at a large referral hospital in Uganda: A prospective observational pilot study
Source: PLoS One. 2025 Sep 3;20(9):e0331512. doi: 10.1371/journal.pone.0331512 (PMC12407487; doi:10.1371/journal.pone.0331512)
Supplement: Attachment — Submitted filename: Response to Reviewers.docx [file pone.0331512.s015.docx]

Bergen, 11 June 2025

**Re: Response to comments from the initial PLOS ONE review of our manuscript:**

*Severe postpartum haemorrhage at a large referral hospital in Uganda: a prospective observational study*

Thank you very much for your input and insightful comments. We have reviewed and addressed the comments in the table on the next page.

Several of the comments pertain to the hypothesis, sample size and assessment of statistical significance and we wanted to make a general comment on this before diving into each of the comments from reviewers and editors.

The aim of the study was to map the occurrence and profile of severe PPH as well as the current challenges faced in its management at Kawempe National Referral Hospital. The prior estimate was that 60 inclusions would suffice to map the occurrence and profile of PPH and the current challenges faced in the management of severe PPH. The aim was to follow up this study with an intervention study including staff training with simulation exercises. But you need to have a rough idea of what the problems are before you can do that.

Initially we had planned to just present the data as a single group, but in the analysis phase we realised the stark difference between the referral and inhouse patients and that is why we presented them as two groups. However, the number of inclusions remains modest and we therefore presented the data without any statistical significance tests, knowing that with as modest number there would be no significances to present.

We now need your view on how to best present the data:

1) Rather merge the two groups (in-house and referrals) into one single group. We have not done this in this submission, but we are prepared to do so, should you require.
2) Present the data as two groups as we have initially done but stating that none of the comparisons were statistically significant. We have done this in the manuscript version A, attached.
3) Present the data with p-value, OR and CI calculations per your recommendation, but due to the small sample size, all but one variable is not statistically significant. We have done this in the manuscript version B, attached.

Let us know which of the versions that are most suitable for your journal. Do not hesitate to contact me, should there be any queries.

Best regards,

Dr Mia Appelbäck

Centre for International Health

University of Bergen, Norway

Mobile: +46 702682661

|  | **Comments** | **Response** | **Line** |
| --- | --- | --- | --- |
|  | **Reviewer 1** |  |  |
| 1. | In line 6, the abbreviation DHS is mentioned for the first time without explanation. To ensure the readership understands its meaning, I recommend defining this acronym in its first use (e.g., Demographic and Health Survey). | Done | Version A: 103  Version B: 96 |
| 2. | Route of Misoprostol Administration: The manuscript references the use of misoprostol, but it is unclear which route of administration was employed — buccal, vaginal, or rectal. Given that the route of administration may significantly influence the drug’s efficacy and absorption rate, I suggest providing this detail. | In the Ugandan national guidelines as a first response bundle, misoprostol should be given rectally or sublingually. As we are looking at whether the guidelines were followed we chose not to separate the two but instead clarify under variables in the methods section that “Misoprostol was administered rectally and/or sublingually” | Version A: 260  Version B: 279 |
|  | **Reviewer 2** |  |  |
| 1. | Abstract: Background: Does not provide the rationale for the study | The following has been added: “Little is known about the outcomes and management of severe PPH at KNRH”.  *The length of the abstract is now 313 (version A) and 327 (version B) respectively once all the changes to the abstract were made.* | Version A: 30-31  Version B: 30-31 |
| 2. | Abstract: Methods: should clearly show the study period and summary of the analysis | The methods section has been changed to include the requested information. | Version A: 34-38  Version B: 34-38 |
| 3. | Introduction: Should contain the global MMR of the post-SDG era (decreeing, increasing, or stagnated?) | The latest MMR from the post-SDG era have now been used and the text changed to the following: “The global maternal mortality ratio (MMR) decreased by over 40% from 2000 to 2023. Yet, an estimated 260 000 women still die each year, with postpartum haemorrhage (PPH) as the leading direct cause” | Version A: 97-99  Version B: 90-92 |
| 4. | Methods:  Participants and procedure: If the recruitment period was from 5th April to 30th May, approximately two months, the data collection period should be more than two months. Because the mother who was recruited on 30th May will be studied again after 42 days. Therefore, the data collection time is more than two months. | Data collection has been changed to recruitment period as, as you point out, the data collection period extended beyond this 2-month period. | Version A: 184-185  Version B: 202-204 |
| 5. | Methods:  Participants and procedure: Why were the minors excluded from the study? | Minors in this context are referred to as emancipated minors and were included in the study. | Version A: 182  Version B: 201 |
| 6. | Methods:  Data collection tools and procedures: Instead of “participants and procedures”, I would recommend “data collection tools and procedures.” | This has been changed accordingly | Version A: 180  Version B: 199 |
| 7. | Methods:  Data collection tools and procedures: Additionally, they have to show in this section that they used EpiData during data collection | “All data were captured on paper and doubly entered in EpiData 3.1 (<http://www.epidata.dk>), verified and merged into one dataset.» was moved from *quantitative variables and statistical methods* to *data collection tools and procedures*. | Version A: 205-206  Version B: 224-225 |
| 8. | Methods:  Sample size: The justification for the sample size to be 60 is not satisfactory. | The aim of the study was to map the occurrence and profile of severe PPH as well as the current challenges faced in its management at KNRH and we did not test a specific hypothesis. The prior assessment was that 60 inclusions would suffice to map the occurrence and profile of PPH and the current challenges faced in the management of severe PPH.  This study was a baseline study for a larger RCT which is why we chose to limit the number of inclusions to 60 in this study.  The text remains unchanged. |  |
| 9. | Methods:  Data quality assurance: There is no evidence of efforts to ensure data quality | Initial quality control of the CRFs was done by a RA, then two senior researchers (MA and CL) checked the data for completeness and correctness. | Version A: 207-208  Version B: 226-227 |
| 10. | Methods:  The statistical methods: Lacks specific details. What type of descriptive statistics have they used? | Please see our initial comment. We are providing two versions of the manuscript, version A with a general statement that there were no significant differences between the two groups and version B where all the differences shown not to be significant. | Version A: 278-279  Version B: 300-307 |
| 11. | Results: Should describe if there were non-responses and why. | “There were no non-responses» has been added to the results section | Version A: 303-304  Version B: 329-330 |
| 12. | Results: Remove the name of the figures in the body of the manuscript, e.g., on line 249, 263 and so on | We have followed the PLOS ONE guidelines for figures, which states Under captions: “**Place** figure captions in the manuscript text in read order, immediately following the paragraph where the figure is first cited. Do not include captions as part of the figure files or submit them in a separate document.” (https://journals.plos.org/plosone/s/figures) |  |
| 13. | Discussion: Implications of the study on maternal and child health practice and policy should be boldy discussed | The final paragraph discusses many issues with implications for maternal health practice and policy but to further highlight the issue the following sentence has been added: “Strengthening the referral system and improving implementation of evidence-based guidelines such as non-pharmaceutical interventions in PPH management should inform maternal health practice and policy» | Version A: 499-501  Version B: 527-529 |
| 14. | Discussion: Remove the word “summary” on line 301 | This has been changed accordingly |  |
|  | **Additional Editor Comments** |  |  |
| 1. | Abstract:  Results: "All the uterine ruptures and 12 out of 13 both stillbirths and hysterectomies were among referrals." Please rewrite this. This is not clear | In the abstract, the wording in now changed to the following: “All the participants with uterine ruptures and 12 out of the 13 participants with stillbirths and hysterectomies, respectively, were among referrals.” | Version A: 42-44  Version B: 42-45 |
| 2. | Abstract:  The results section fails to show level of significance. This has to be revised as referrals vs non-referrals with 95% CI and p-value. | Please see comment 10, reviewer 2. | Version A: -  Version B: 44-64 |
| 3. | Abstract:  Suboptimal wellbeing is not a medical term. What do you mean by suboptimal wellbeing? | Suboptimal is defined as fair/poor wellbeing in our results and this has been added in the abstract and elaborated in methods and in table 4. | Version A: 48 + 243-244  Version B: 63 + 262-263 |
| 4. | Introduction: The data you used for the global maternal mortality is old. Please use recent reports by the interagency group released in 2025. | This has been rectified, see also point 3, reviewer 2. | Version A: 97-99  Version B: 90-92 |
| 5. | Methods: The justification put forth for the sample size is not scientific. It does not matter if your study is descriptive or analytic an appropriate sample size should be sought. | Please see comment 8, reviewer 2. |  |
| 6. | Results: Could you please calculate the p-value for each variable being compared in between the two groups to see the level of significance of the differences? | Please see comment 10, reviewer 2. | Version A: -  Version B: Table 1-4 |
| 7. | Discussion: please remove 'summary' | This has been removed accordingly |  |
| 8. | First paragraph: start with restating your objective, and then follow summarizing the main findings that you will subsequently discuss. | The discussion now starts with the objective. | Version A: 390-391  Version B: 418-419 |
| 9. | Most of the graphs are not necessary and they can be described within text. | We have removed all the graphs except the two under “key clinical indicators”, knowing that graphs are excellent entry points to generate readers interest. | Version A: 321-345  Version B: 349-368 |
