# Supplementary material for: Severe postpartum haemorrhage at a large referral hospital in Uganda: A prospective observational pilot study
Source: PLoS One. 2025 Sep 3;20(9):e0331512. doi: 10.1371/journal.pone.0331512 (PMC12407487; doi:10.1371/journal.pone.0331512)
Supplement: Attachment — Submitted filename: Rebuttal letter_250727.docx [file pone.0331512.s016.docx]

Bergen, 27 July 2025

**Re: Response to comments from the second PLOS ONE review of our manuscript:**

*Severe postpartum haemorrhage at a large referral hospital in Uganda: a prospective observational pilot study*

Thank you for your input and comments. We have addressed the comments in the table below.

Best regards,

Dr Mia Appelbäck

Centre for International Health

University of Bergen, Norway

Mobile: +46 702682661

|  | **Comments** | **Response** | **Line** |
| --- | --- | --- | --- |
| 1. | Please state that this is a pilot study in the title, in the abstract, and the main body and state that a large RCT will be conducted | It has been added that it is a pilot study in the title and abstract and the following sentence has been added to the methods section: “It was a pilot study for a large randomised control trial.” | 3, 34, 111 |
| 2. | Please use option A for the resubmission | Done |  |
